# *Clostridium difficile* Infection Associated with Pig Farms

**DOI:** 10.3201/eid1906.121645

**Published:** 2013-06

**Authors:** Elisabeth C. Keessen, Céline Harmanus, Wietske Dohmen, Kuijper, Len J.A. Lipman

**Affiliations:** Utrecht University, Utrecht, the Netherlands (E.C. Keessen, W. Dohmen, L.J.A. Lipman);; University Medical Center, Leiden, the Netherlands (C. Harmanus, E.J. Kuijper)

**Keywords:** Clostridium difficile, pigs, farmers, pig farms, workers, agriculture, livestock, zoonoses, enteric infections, bacteria

**To the Editor:** *Clostridium difficile* of PCR ribotype 078 causes enteric disease in humans and pigs (*1*,*2*); a recent pan-European study revealed that this type was the third most frequently found type of *C. difficile* (*1*). The finding of identical *C. difficile* PCR ribotype 078 isolates in piglets with diarrhea and in humans with *C. difficile* infection (CDI) led to the suggestion that interspecies transmission might occur (*3*,*4*). Because *C. difficile* can be detected in the immediate environment of pig farms, we investigated intestinal colonization with *C. difficile* in pigs and in pig farmers, their relatives, and their employees in the Netherlands.

Persons living on 32 pig farms were enrolled as part of a longitudinal intervention study of several zoonotic agents. Pig farmers were partly recruited through the Dutch Farmer’s Association or by veterinarians, who informed potential participants about the aims of the study. Inclusion criteria for participants were that they should work and/or live on the farm; the farms were either closed farms or multipliers (farms at which piglets are bred and then sold to other farms, where they are raised until ready for slaughter). The number of persons willing to submit a fecal sample per farm ranged from 1 to 10 (mean 4, median 5). Veterinarians who normally provided veterinary services to each farm collected fresh fecal samples from the floors of 10 animal wards per farm. No a priori knowledge of *C. difficile* colonization status of the pigs on the farms was available. Fecal samples from humans and from animal wards were cultured for the presence of toxinogenic *C. difficile* by using previously described methods (*1*,*3*,*4*).

Of the 128 persons who enrolled in the study, 48 had daily contact with pigs, 22 had weekly contact with pigs, and 36 had contact with pigs varying from monthly to less than yearly; no contact information was available for 22 participants. A total of 12 (25%) of 48 persons who had daily contact with pigs had fecal samples positive for *C. difficile* colonization; for persons who had weekly contact with pigs, 3 (14%) of 22 had positive samples. Daily to weekly contact with pigs versus monthly to less than yearly contact was significantly associated with an intestinal presence of *C. difficile* (p = 0.003). *C. difficile* was also found in fecal samples from 3 persons for whom no contact information was available. The *C. difficile* carriage rate among those with daily to weekly contact with pigs (15/70, 21%) was higher than the carriage rate of <5% reported for nonhospitalized adults with CDI (*5*).

A total of 18 *C. difficile*–positive human samples were detected at 16 of 32 pig farms investigated. At 2 of these farms, only 1 person submitted a sample, but at the other 14 farms, the number of participants ranged from 2 to 9 (mean 4, median 3). *C. difficile* was found in pig manure at all farms; 10%–80% of the wards were positive per farm. 

Corresponding *C. difficile* PCR ribotypes were cultured from samples from pigs and humans; type 078 was found in humans and pigs on 15 farms and type 045 in a farmer and his pigs on 1 farm. Multilocus variable number tandem repeat analysis (MLVA) and antimicrobial drug susceptibility testing (E-test) were performed on human isolates from 15 farms and 1 porcine isolate per farm. One human isolate could not be typed because the isolate was lost during laboratory activities.

MLVA results showed that, at 2 farms, the human and porcine isolates were not genetically related, whereas at the other 13 farms, human and porcine isolates were genetically related, including 100% identical MLVA results for type 078 human and porcine isolates at 3 farms. Isolates were considered genetically related when the summed tandem repeat differences were <10 (*3*,*4*,*6*). 

Antimicrobial drug susceptibility testing demonstrated similar susceptibility levels among isolates. For human and porcine isolates from 9 of 15 farms, MIC variability of <1 μg/L was found for imipenem, cotrimoxazole, erythromycin, clindamycin, tetracycline, and moxifloxacin. For the remaining 6 farms, drug susceptibility patterns for human isolates differed from pig isolates for 1 drug only: for 1 farm, MICs of erythromycin were 256 μg/L for human isolates and 0.38 μg/L for pig isolates; for 3 farms, MICs of erythromycin were 256 μg/L for pig isolates and 0.25 μg/L for human isolates; and for 2 farms, MICs of imipenem were 32 μg/L for pig isolates and 1.5 or 2 μg/L for human isolates. 

In summary, the high *C. difficile* carriage rate among persons who had direct contact with pigs and the fact that these *C. difficile* isolates were genotypically and phenotypically similar to the pig isolates from the same farms indicates that transmission occurs either by direct contact or through the environment. Prospective studies are needed to determine the relationship between *C. difficile* carriage and development of CDI in this population.
